# Risk factors for differential outcome following directly observed treatment (DOT) of slum and non-slum tuberculosis patients: a retrospective cohort study

**DOI:** 10.1186/s12879-016-1835-1

**Published:** 2016-09-20

**Authors:** Robert E. Snyder, Mariel A. Marlow, Melissa E. Phuphanich, Lee W. Riley, Ethel Leonor Noia Maciel

**Affiliations:** 1 Division of Infectious Diseases and Vaccinology and Division of Epidemiology, School of Public Health, University of California, Berkeley, 94720 CA USA; 2 Rush Medical College, 600 S Paulina St, Chicago, 60612 IL USA; 3 Laboratory of Epidemiology, Universidade Federal do Espírito Santo, Avenida Marechal Campos, 1468 Maruípe, Vitória, ES Brazil

**Keywords:** Tuberculosis treatment outcome, Directly observed treatment, Tuberculosis cure, Urban poverty, Slum

## Abstract

**Background:**

Brazil’s National Tuberculosis Control Program seeks to improve tuberculosis (TB) treatment in vulnerable populations. Slum residents are more vulnerable to TB due to a variety of factors, including their overcrowded living conditions, substandard infrastructure, and limited access to healthcare compared to their non-slum dwelling counterparts. Directly observed treatment (DOT) has been suggested to improve TB treatment outcomes among vulnerable populations, but the program’s differential effectiveness among urban slum and non-slum residents is not known.

**Methods:**

We retrospectively compared the impact of DOT on TB treatment outcome in residents of slum and non-slum census tracts in Rio de Janeiro reported to the Brazilian Notifiable Disease Database in 2010. Patient residential addresses were geocoded to census tracts from the 2010 Brazilian Census, which were identified as slum (*aglomerados subnormais* -AGSN) and non-slum (non-AGSN) by the Census Bureau. Homeless and incarcerated cases as well as those geocoded outside the city’s limits were excluded from analysis.

**Results:**

In 2010, 6,601 TB cases were geocoded within Rio de Janeiro; 1,874 (27.4 %) were residents of AGSN, and 4,794 (72.6 %) did not reside in an AGSN area. DOT coverage among AGSN cases was 35.2 % (*n* = 638), while the coverage in non-AGSN cases was 26.2 % (*n* = 1,234). Clinical characteristics, treatment, follow-up, cure, death and abandonment were similar in both AGSN and non-AGSN TB patients. After adjusting for covariates, AGSN TB cases on DOT had 1.67 (95 % CI: 1.17, 2.4) times the risk of cure, 0.61 (95 % CI: 0.41, 0.90) times the risk of abandonment, and 0.1 (95 % CI: 0.01, 0.77) times the risk of death from TB compared to non-AGSN TB cases not on DOT.

**Conclusion:**

While DOT coverage was low among TB cases in both AGSN and non-AGSN communities, it had a greater impact on TB cure rate in AGSN than in non-AGSN populations in the city of Rio de Janeiro.

## Background

Between 1990 and 2012, the incidence of tuberculosis (TB) in Brazil decreased by an average of 1.3 % each year. Despite this decrease, the country remains one of twenty-two World Health Organization (WHO) high-burden TB countries [1]. Brazil also has a large urban slum population (as defined by both the United Nations and the Brazilian Census Bureau), where 11.4 million (6.0 %) of its roughly 200 million people reside. Rio de Janeiro has the nation’s largest slum population, with 1.4 million of the city’s 6.3 million people (22 % of the population) residing in these communities [2].

The Sistema de Informação de Agravos de Notificação (SINAN) is Brazil's national Notifiable Disease Surveillance System to which all TB cases are compulsorily reported. The proportion of incident TB cases detected by SINAN ranges from 85 to 90 % [1].

The Stop TB Partnership’s Global Plan to Stop TB has as its primary goals to address HIV-related TB, limit the spread and development of multidrug-resistant (MDR) TB, and empower communities and vulnerable populations afflicted with TB [3]. To this end, in 2006 Brazil augmented its National TB Control Program (NTCP) by expanding the use of directly observed treatment (DOT) and enhancing laboratory diagnostic capabilities [4].

In 2010, the burden of TB (measured in disability-adjusted life years - DALY) was higher among residents of Rio’s slums than among its non-slum residents [5]. While previous work has shown that DOT has been effectively implemented in vulnerable populations in Brazil [6], a comparative analysis of the impact of DOT in slums and formal communities has yet to be done. This study examines the impact of DOT on TB treatment outcome in these two types of neighborhood in the city of Rio de Janeiro, Brazil.

## Methods

This is a retrospective analysis of all TB cases (7,276) with onset in 2010 that were reported to SINAN in Rio de Janeiro, Brazil. The cases were geocoded by patient residential address with Google Geocoding API v.3 (https://developers.google.com/maps/documentation/geocoding/start) and mapped in ArcGIS 10 (ESRI, Redlands, CA, USA). TB patients whose residences were outside of the city limits (142, 2.0 %), who were incarcerated (452, 6.2 %), or were homeless (81, 1.1 %) were excluded.

The 2010 Census classified a census tract as “*aglomerados subnormal*” or AGSN if the tract included a portion (or all) of a community of at least 51 homes that illegally occupied the land (i.e. was constructed on the property of others) or received a land title in the previous ten years) and at least one of the following: 1) a general scarcity of public services or 2) construction outside of existing municipal patterns [2]. TB cases in the SINAN database were overlaid and mapped on the census tract shapefiles from the 2010 Brazilian Census. Individuals with TB who lived in or within 50 meters of an AGSN census tract were identified as slum TB cases.

Demographic (age, sex), clinical characteristics (pulmonary or extrapulmonary, relapse or not), TB diagnostic results (tuberculin skin test and acid-fast bacilli smear results), treatment (DOT, time from diagnosis until notification) and follow-up (contact tracing) were compared between slum (AGSN) and non-slum (non-AGSN) TB cases in the city. Analyses were performed in Stata 12.1 (Statacorp, College Station, USA).

TB treatment outcomes were consistent with the Brazilian Ministry of Health’s definitions [7]. Cure was defined as two consecutively negative sputum smears, one before and one after cessation of chemotherapy. Treatment abandonment was defined as a patient’s absence from the treatment center for a minimum of 30 days after the return date indicated by his or her health professional. TB treatment outcomes (including mortality) are reported to and updated in the SINAN database by health professionals at the health unit where treatment occurs.

Health professionals at the primary health units where suspected TB patients are first encountered report all new TB cases to SINAN using a standardized case notification form. These suspected cases are prospectively followed until the case is considered closed with one of the following outcomes: cure, abandonment, death from TB, death from other cause, transfer, or missing outcome.

In Brazil, DOT is implemented in accordance with WHO recommendations [8], by the country’s primary health system (Sistema Único de Saúde - SUS). The patient appears at the treatment center a minimum of three times each week during the first two months of treatment, or has the same frequency of home visits by community health workers [7]. The Brazilian Ministry of Health’s policy is that all TB cases (new and reactivation TB) should receive DOT. In practice, health professionals use DOT for patients perceived as being at greatest risk of treatment failure. The decision to observe treatment at home or at the health clinic is made by the health professional in consultation with the patient. This policy also alludes to the importance of making this decision given existing limitations in infrastructure or availability of human resources (e.g. if there are enough employees to observe treatment, or if a health unit is geographically proximal to the patient) [7]. In Brazil, provision of food and transportation vouchers for those enrolled in DOT is at the discretion of individual health professionals and encouraged by the Ministry of Health when a professional believes it will enhance treatment adherence [9].

We assessed differences in the demographic and socioeconomic characteristics of TB cases in AGSN and non-AGSN areas using the Mantel-Haenszel (MH) chi-squared test and Students T-test where appropriate. We calculated relative risks (RR) with the Mantel-Haenszel (MH) chi-squared test for treatment outcomes (e.g. death, cure rate, abandonment, transfer, etc.) in those who did or did not undergo DOT to compare treatment outcomes of TB cases on DOT versus treatment outcomes of TB cases not on DOT.

After this crude analysis, multivariable logistic regression models were developed to adjust for other factors that may have influenced the relationship between DOT, AGSN residence, and TB treatment outcomes: cure, abandonment, and death from TB. Those who transferred elsewhere for treatment (590, 8.9 %) or were missing treatment outcome data (688, 10.4 %) were excluded from all models, leaving 5,266 (80.7 %) eligible TB cases. Potential confounding variables were included if they were related to TB treatment outcome. Those available in SINAN include: age, sex, HIV status, alcoholism, diabetes, relapse (a TB case that was previously declared cured), and disease severity (extrapulmonary v. pulmonary), in addition to DOT status, and residence in an AGSN [10–12]. Models were created in a forward stepwise process, maintaining covariates in the final models with p-values less than 0.20. Dummy variables were created to test for interaction between age and sex, as well as DOT and residence in a slum. Using these dummy variables, we evaluated the hypothesis that DOT treatment varied in effectiveness between AGSN and non-AGSN TB cases [13, 14].

## Results

In 2010, there were 1,807 TB cases among residents of AGSN areas (0.13 % of the total AGSN population, 27.4 % of all TB cases in Rio de Janeiro in 2010), and 4,794 cases among residents of non-AGSN areas in Rio de Janeiro (0.10 % of the total non-AGSN population, 76.2 % of 2010 Rio TB cases) that were reported to SINAN (Table 1). Among all covariates, the average proportion of missing covariates for eligible participants was 0.21 %. Of 6,601 total TB cases, 2,317 (35.1 %) were in women. The mean age of all TB cases was 38.7 years (standard deviation: 16.5).

**Table 1:** Slum (*aglomerados subnormais* - AGSN) and non-slum (non-AGSN) tuberculosis clinical and treatment characteristics, Rio de Janeiro, Brazil, 2010

	Total n (%)	Slum n (%)	Non-slum n (%)	*P*-value^*^
Number of cases	6601 (100.0)	1807 (27.4)	4794 (72.6)	-
Median age (IQR)	37 (26, 50)	38 (37, 51)	33 (23, 47)	-
Sex (% female)	2317 (35.1)	1666 (34.8)	651 (36.0)	0.313
Site of TB				
Pulmonary	5412 (82.0)	1515 (83.8)	3897 (81.3)	0.02
Extrapulmonary	867 (13.1)	218 (12.1)	649 (13.5)	0.11
Pulmonary and extrapulmonary	322 (4.9)	74 (4.1)	248 (5.2)	0.07
Case category				
New case	5173 (78.4)	1362 (75.4)	3811 (79.5)	<0.001
Relapse after previous treatment completion	483 (7.3)	167 (9.2)	316 (6.6)	<0.001
Relapse after loss to follow-up in previous treatment	545 (8.3)	177 (9.8)	368 (7.7)	0.01
Unknown	73 (1.1)	16 (0.89)	57 (1.2)	0.29
Transfer to other municipality	327 (5.0)	85 (4.7)	242 (5.1)	0.57
Tuberculin skin test result				
Not reactive (0-4 mm)	266 (4.0)	57 (3.2)	209 (4.4)	0.03
Weak reaction (5-9 mm)	101 (1.5)	33 (1.8)	68 (1.4)	0.23
Strong reaction (≥10 mm)	638 (9.7)	175 (9.7)	463 (9.7)	0.97
Not performed	5590 (84.7)	1542 (85.3)	4048 (84.4)	0.37
Acid-fast bacilli smear at diagnosis				
Positive	3228 (48.9)	959 (53.1)	2269 (47.3)	<0.01^§^
Negative	1551 (23.5)	368 (20.4)	1183 (24.7)	
Not performed	1822 (27.6)	480 (26.6)	1342 (28.0)	
Indicated for DOT at diagnosis				
Yes; completed full course	1892 (28.7)	636 (35.2)	1256 (26.2)	<0.01^†^
Yes; did not complete full course	10 (0.2)	10 (0.6)	-	
No	4410 (66.8)	1081 (59.8)	3329 (69.4)	
Unknown	289 (4.4)	80 (4.4)	209 (4.4)	
Treatment changed due to intolerance or failure				
Yes	45 (0.7)	17 (0.9)	28 (0.6)	0.12
No/not recorded	6556 (99.3)	1709 (99.1)	4766 (99.4)	
All contacts indicated at diagnosis examined at follow-up				
Yes	3349 (51.2)	855 (47.8)	2494 (52.5)	<0.01
No	3195 (48.8)	935 (52.2)	2260 (47.5)	
Median days (IQR) from diagnosis until notification	0 (0, 14)	0 (0, 14)	1 (0, 14)	-
Median days (IQR) from diagnosis until initiation of treatment	0 (0,0)	0 (0, 0)	0 (0, 1)	-
Median days (IQR) from initiation of treatment until change of treatment due to adverse reaction	175 (77, 238)	182 (79.5, 237.5)	149 (76, 244)	-
Median days (IQR) from diagnosis until end of follow-up	187 (145, 219)	187 (146, 218)	189 (145, 221)	-

*IQR* interquartile range, 25th and 75th percentile

^*^ *P*-value calculated to compare selected characteristics between cases in slums versus and non-slums

^§^Comparing positive versus negative AFB smear diagnosis

^†^Comparing all those assigned to DOT (regardless of completion)

Of the 1,807 cases living in AGSN areas 638 (35.2 %) were on DOT while 1,234 (26.2 %) TB patients living in non-AGSN areas were on DOT. Among TB patients living in AGSN areas, 963 (56.7 %) patients were cured, while 2,622 (57.8 %) TB patients living outside of AGSN areas were cured (Table 2). Of 1,807 AGSN TB patients, 325 (19.1 %) abandoned treatment, and 746 (16.5 %) non-AGSN TB patients did so (Table 2). Among AGSN TB patients, 32 (1.9 %) died from other causes while 68 (4.0 %) died from TB. Of the non-AGSN cases, 128 (2.8 %) died from other causes while 199 (4.4 %) died from TB. Among AGSN TB cases, 17 (2.7 %) on DOT died of all causes, while among non-AGSN cases 52 (4.2 %) not on DOT and 275 (8.4 %) on DOT died of all causes.

**Table 2:** Comparison of tuberculosis treatment outcome between patients receiving and not receiving directly observed treatment, separated by census tract of residence: slum (*aglomerados subnormais -* AGSN) and non-slum (non-AGSN), Rio de Janeiro, Brazil, 2010

	AGSN					Non-AGSN					*P*-value^†^
	DOT (%)			RR^§^	95 % CI	DOT (%)			RR^§^	95 % CI	
	Total	Yes	No			Total	Yes	No			
Missing outcome	174 (10.2)	39 (6.1)	135 (12.7)	0.56	0.42-0.74	462 (10.2)	98 (7.9)	364 (11.0)	0.77	0.64-0.92	0.06
Cure	963 (56.7)	430 (67.4)	533 (50.1)	1.66	1.45-1.91	2622 (57.8)	758 (61.4)	1864 (56.5)	1.15	1.04-1.27	<0.01
Abandonment	325 (19.1)	118 (18.5)	207 (19.5)	0.97	0.83-1.14	746 (16.5)	265 (21.5)	481 (14.6)	1.37	1.23-1.53	<0.01
Death TB	68 (4.0)	8 (1.3)	60 (5.6)	0.20	0.08-0.46	199 (4.4)	28 (2.3)	171 (5.2)	0.54	0.38-0.76	0.03
Death other	32 (1.9)	9 (1.4)	23 (2.2)	0.68	0.37-1.24	128 (2.8)	24 (1.9)	104 (3.2)	0.65	0.45-0.95	0.92
Transfer out	140 (8.2)	34 (5.3)	106 (10.0)	0.57	0.41-0.78	378 (8.3)	61 (4.9)	317 (9.6)	0.60	0.47-0.75	0.82

^*^There were 88 (4.4 %) cases in AGSN and 219 (4.4 %) outside of AGSN where the case’s DOT status was unknown

^§^Relative risk (RR) estimating crude risk of outcome in patients undergoing DOT compared to patients not undergoing DOT in slum versus non-slum neighborhood

^†^ *P*-values assessing for effect measure modification of AGSN census tracts on the relationship between DOT and respective outcome with the Mantel-Haenszel chi-square test

Unadjusted bivariate analyses indicated that those living in AGSN and on DOT were 1.66 (95 % confidence interval (CI): 1.45-1.91) times as likely to be cured and had 0.20 (95 % CI: 0.08-0.46) times the risk of death compared to those not on DOT in AGSN. Outside of AGSN, those on DOT had 1.15 (95 % CI: 1.04-1.27) times the probability of cure and 0.54 (95 % CI: 0.38-0.76) times the risk of death compared to those not on DOT. Of those on DOT in AGSN, 118 (18.5 %) abandoned treatment, while 265 (21.5 %) non-AGSN patients did so. Those who transferred to another municipality for TB treatment or who were missing their treatment outcome were not more likely to be residents of an AGSN.

Holding other factors constant (age, sex, HIV/AIDS and clinical disease presentation - pulmonary v. extrapulmonary), TB cases residing in AGSN areas were less likely to be cured (0.77, 95 % CI: 0.63-0.95) than TB cases living outside of AGSN areas (Table 3). TB cases on DOT and living outside of AGSN were 1.16 times as likely to be cured (95 % CI: 0.95-1.42) as TB cases not on DOT living outside of AGSN. However, if the TB case was a resident of an AGSN area and on DOT, the chance of cure increased to 1.67 (95 % CI: 1.17-2.40) compared to TB cases that were not residents of AGSN and not on DOT.

**Table 3:** Logistic regression models predicting risk of cure (model 1), abandonment of treatment (model 2) and death (model 3) from tuberculosis among all TB cases in Rio de Janeiro, Brazil, 2010

	Odds ratio	Standard error	95 % confidence interval
Model 1: risk of cure^*^			
On directly observed treatment (DOT)	1.16	0.12	0.95, 1.42
Residence in a slum (AGSN) area	0.77	0.08	0.63, 0.95
Sex (reference female)	0.73	0.06	0.62, 0.85
Age in years	1.01	0.002	1.00, 1.01
Extrapulmonary clinical disease	2.07	0.23	1.66, 2.59
HIV/AIDS	0.23	0.02	0.19, 0.28
DOT x AGSN (interaction term)	1.67	0.31	1.17, 2.40
Model 2: risk of abandonment^§^			
On directly observed treatment (DOT)	1.15	0.13	0.92, 1.43
Residence in a slum (AGSN) area	1.33	0.16	1.05, 1.67
Sex (reference female)	1.41	0.13	1.18, 1.69
Age in years	0.98	0.003	0.97, 0.98
Extrapulmonary clinical disease	0.44	0.06	0.33, 0.57
HIV/AIDS	1.97	0.23	1.56, 2.48
DOT x AGSN (interaction term)	0.61	0.12	0.41, 0.90
Model 3: risk of death^†^			
On directly observed treatment (DOT)	0.39	0.11	0.23, 0.67
Residence in a slum (AGSN) area	1.23	0.23	0.85, 1.78
Age in years	1.03	0.01	1.02, 1.04
Extrapulmonary clinical disease	0.64	0.13	0.43, 0.95
HIV/AIDS	8.79	1.45	6.35, 12.16
DOT x AGSN (interaction term)	0.10	0.10	0.01, 0.77

*AGSN* aglomerados subnormais

^*^N: 3797, R^2^ = 0.061

^§^N: 3797, R^2^ = 0.047

^†^N: 3797, R^2^ = 0.175

The risk of treatment abandonment was similarly influenced by DOT and the TB case’s location of residence. Controlling for sex, age, clinical disease severity, and HIV/AIDS, we found that TB patients that resided in an AGSN were 1.33 times more likely to abandon treatment (95 % CI: 1.05-1.67) (Table 3) than TB patients not residing in an AGSN. Being on DOT did not have a statistically significant effect on risk of treatment abandonment (1.15; 95 % CI: 0.92-1.43). However, TB cases residing in an AGSN area and on DOT had 0.61 times the risk of abandonment (95 % CI: 0.41-0.90) compared to TB cases not in AGSN and not on DOT.

Similarly, the relationship between a TB case being on DOT and dying from TB was also modified by whether or not the case resided in an AGSN area (Table 3). When controlling for age, clinical disease severity, and HIV/AIDS, being on DOT decreased the risk of death from TB by a factor of 0.39 (95 % CI: 0.23-0.67). Being a resident of an AGSN did not significantly increase the risk of death from TB (1.23, 95 % CI: 0.85, 1.78). Being on DOT and residing in an AGSN area reduced the risk of death from TB by a factor of 0.1 (95 % CI: 0.01, 0.77) compared to not being on DOT and not residing in an AGSN.

## Discussion

Slum-defining characteristics, such as overcrowding, poor access to healthcare, and poverty are associated with worse TB treatment completion and disease outcomes [15, 16]. Here, we found that DOT administered to TB patients living in Rio de Janeiro, Brazil's informal settlements (AGSN) lowered a TB cases’ risk of treatment abandonment, or death from TB, and increased the chance of being cured. The Brazilian NTCP makes explicit its prioritization of DOT in vulnerable populations, but has yet to adapt these policies to residents of slums. The differential effectiveness of DOT inside and outside of AGSN in Rio de Janeiro highlighted here, lends support to the hypothesis that residents of slums should be considered a vulnerable population when considering DOT as a treatment strategy for TB.

In Rio de Janeiro in 2010, DOT coverage was higher among AGSN TB cases (35.2 %) than in non-AGSN TB cases (26.2 %). Cure rates, however, remained low in both communities. The TB cure rates inside (56.7 %) and outside of AGSN (57.8 %) were nearly identical, and roughly the same as the 2010 Brazilian average (61.9 %), which is well below the Millennium Development Goal of 85 % cure [1]. However, patients on DOT residing in AGSN areas had better outcomes than patients on DOT outside of AGSN areas. Within AGSN areas, TB cases were 1.67 (95 % CI: 1.17-2.40) times more likely to be cured if on DOT, compared to cases not on DOT and not living in AGSN areas. In AGSN, the proportion of deaths due to TB was 4.3 times higher among those who were not on DOT, while it was 2.3 times higher in the non-AGSN population not on DOT (Table 2).

Although treatment abandonment was higher in AGSN than non-AGSN patients, abandonment by those on DOT was less likely among TB patients living in AGSN areas (18.5 %) than among TB patients not living in AGSN areas (21.5 %). The higher rates of treatment abandonment among those on DOT may be biased by the program’s design, as it is strongly encouraged as a treatment option for those considered at risk for abandoning treatment.

Importantly, the provision of food and transport vouchers is not noted in the SINAN database (a practice encouraged to reduce abandonment). A 2015 survey of health professionals from five different regions in Brazil (not including Rio de Janeiro) indicated that only 51 % and 20.5 % of surveyed clinics had access to food and transport vouchers, respectively. It is possible that these vouchers are more frequently given to TB cases that reside in slums to discourage their risk of treatment abandonment while on DOT, but we cannot evaluate this bias due to the absence of these data in SINAN [9].

Almost 5 % (289) of the SINAN database is lacking data on DOT. In contrast to the observational data presented here, a 2015 Cochrane meta-analysis of DOT randomized control trials found questionable improvements in TB outcome, highlighting the need to indicate where DOT is administered (home or health facility) and who observes treatment (family member or health professional) [17]. Completing these missing data, including the 10 % of cases that were missing data on treatment outcome, and simultaneously augmenting the SINAN database by including location of DOT, and who is observing treatment could further clarify how DOT works.

Of more concern is the fraction of data that are missing for other important covariates. Almost a quarter of the 2010 TB cases (25.63 %) did not have their HIV status recorded in SINAN despite the fact that HIV status was the most important risk factor for cure, risk, and abandonment. Health professionals must continue to make efforts to identify all HIV-positive TB patients and reliably report their HIV-status to SINAN.

There are several possible explanations for the apparent greater impact of DOT on TB cases in AGSN. The program is overseen by SUS’s community-based health program, in which residents of the community where the primary health centers are located serve as that health center's community-health workers. The familiarity of these agents with their patients and communities could have contributed to the differential success of DOT in AGSN. The quality of DOT coverage, therefore, may have differed between the two populations.

This analysis is based on data from SINAN, which has a high case-detection rate, but that relies on cases being reported [1]. A 2008 study in Rio de Janeiro found that roughly half of the city's TB deaths were not reported to SINAN [18]. This was likely due to the severity of the disease when it was diagnosed (i.e. patients who died and were included in the mortality database but not in SINAN were already at an advanced stage of disease when admitted to the hospital and died before they were reported to SINAN). Inclusion of data from the city’s mortality database in this analysis would have increased the number of deaths, but likely would not have influenced the differential influence of DOT in AGSN.

Case reporting and reliability of the database must be enhanced so that the effectiveness of DOT can be thoroughly evaluated. The differential effect of DOT on TB cases residing in AGSN areas should be confirmed in future analyses as the quality of data reporting in SINAN continues to improve. These findings suggest that DOT should be more thoroughly implemented both inside and outside of slums. The relatively greater effectiveness of DOT within slums further emphasizes the importance of identifying which TB cases reside in slums.

## Conclusions

There were no notable differences in treatment between AGSN and non-AGSN TB cases in Rio de Janeiro. TB treatment outcomes were worse among TB cases not on DOT, with DOT demonstrating the greatest impact on cure rates in vulnerable AGSN communities. Our results suggest an effect of DOT on mortality as well. The Brazilian NTCP’s success with DOT in the AGSN communities of Rio de Janeiro offers an established model for improving TB care in vulnerable slum populations in other global megacities.

## Abbreviations

AGSN: *Aglomerados subnormais*
DOT: Directly observed treatment
MDR-TB: Multi-drug resistant tuberculosis
MH: Mantel-Haenszel
NTCP: National tuberculosis control program
RR: relative risk
SINAN: *Sistema de Informação de Agravos de Notificação*
SUS: *Sistema Única de Saúde*
TB: Tuberculosis
WHO: World Health Organization
